# The Relationship between Mindfulness and Job Burnout of Chinese Preschool Teachers: The Mediating Effects of Emotional Intelligence and Coping Style

**DOI:** 10.3390/ijerph19127129

**Published:** 2022-06-10

**Authors:** Yingjie Wang, Bowen Xiao, Ying Tao, Yan Li

**Affiliations:** 1 School of Teacher Education, Huzhou University, Huzhou 313000, China; 03066@zjhu.edu.cn; 2 Department of Educational and Counselling Psychology, and Special Education, The University of British Columbia, Vancouver, BC V6T 1Z4, Canada; bowen.xiao@ubc.ca; 3 School of Early Childhood Education, Shanghai Normal University, Shanghai 200234, China; sybilna@163.com

**Keywords:** mindfulness, job burnout, emotional intelligence, coping style, preschool teachers

## Abstract

Preschool teachers’ job burnout has many adverse effects on their career development; although some studies have examined the influencing factors of teachers’ burnout, less were explored from the perspective of individual factors. This study aimed to examine the relationship between mindfulness and job burnout of preschool teachers, and the mediating effects of emotional intelligence and coping style. A total of 394 preschool teachers in China filled in questionnaires measuring mindfulness, emotional intelligence, coping style, and job burnout. The findings suggested that: (1) mindfulness was negatively related to job burnout; (2) emotional intelligence and negative coping style played independent mediating effects between mindfulness and job burnout; and (3) emotional intelligence and positive coping style played a chain mediating effect between mindfulness and job burnout. The results revealed the mechanism of mindfulness on preschool teachers’ job burnout, which is of great significance for the psychological intervention of preschool teachers in the future.

## 1. Introduction

Job burnout usually refers to the state of exhaustion in emotion, cognition, and behavior driven by harsh working environment, insufficient job resources, and unhealthy interpersonal relations [1], including three dimensions: emotional exhaustion, low personal accomplishment, and depersonalization [2]. As one of the groups most prone to job burnout, preschool teachers have to face the stress from children’s parents, kindergartens, society, and other aspects on top of the heavy work of nursing and education every day. A study on urban Chinese preschool teachers suggested that 53.2% of preschool teachers are experiencing job burnout [3]. Preschool teachers’ job burnout will not only lead to high emotional stress, low teaching quality, poor interpersonal relations, as well as physical and mental health problems [3,4], but also an important reason for their resignation [5]. Therefore, exploring the mechanism of preschool teachers’ job burnout becomes more necessary. Some empirical studies have found the influential factors of burnout from the perspective of organization and environment, such as job environment [6], job stress [7], and insufficient social support [8], while a few studies from the aspect of individual characteristics (e.g., emotional intelligence and personality) influence teachers’ burnout [9,10]. Among these individual factors, teachers’ mindfulness has been proved closely related to their job burnout, as well as physical and mental health [11]. However, the mechanism of how preschool teachers’ mindfulness reduces their job burnout is still unclear. Accordingly, the present study was to explore the mediating roles of emotional intelligence and coping styles in the association between mindfulness and preschool teachers’ job burnout in Chinese culture.

### 1.1. Mindfulness and Job Burnout

Mindfulness is defined as paying complete attention to the moment-to-moment experience on purpose and without judgment [12]. Mindfulness consists of two parts: self-regulation of attention and orientation to experience. The former refers to attention self-regulation and maintaining experience while constantly focusing on the present moment. The latter refers to a particular attitude toward the current experiences, such as acceptance, curiosity, and openness. Individuals who practice mindfulness are intentionally aware of their own internal and external experiences, fully immersing themselves in current activities without subjectively evaluating or labeling their thoughts and feelings [13]. Preschool teachers may experience less stress and negative feelings if they can perceive and embrace their feelings in their daily life and at work without over-evaluating and labeling their own emotional and physical feelings. Otherwise, they may suffer from increased occupational stress or job burnout [14]. Studies on special education teachers, nurses, and athletes also suggested that teachers’ mindfulness was negatively related to their burnout [10,15,16]. Some mindfulness-based interventions for teachers also revealed that training of mindfulness could significantly reduce their job burnout, depression, and anxiety and improve the level of their mental health [17,18]. In summary, we hypothesized that preschool teachers’ mindfulness would be negatively associated with their job burnout.

### 1.2. Emotional Intelligence as a Mediator

Some researchers combined mindfulness and emotion regulation concepts and proposed the Mindfulness Emotion Regulation Model [19]. According to the model, mindfulness focuses on the individual’s ability to be aware and nonjudgmental. Unlike the traditional emotional model, this model emphasizes awareness and nonreactivity rather than suppression, reappraisal, or change of one’s own emotional experience. Nonjudgmental awareness may promote healthy emotional engagement, allowing individuals to genuinely experience and express their emotions. According to the Mindful Emotion Regulation Model, mindfulness can assist individuals in consciously selecting and identifying thoughts, emotions, and feelings, but it does not generate habitual responses, gradually eliminating the automatic evaluation of troubling emotions [19]. A higher level of mindfulness can improve individuals’ emotion regulation skills, reducing feelings of job burnout. Emotional intelligence may play an important role in the relationship between mindfulness and job burnout. Emotional intelligence (EI) is the ability to recognize and monitor one’s own and others’ emotions, as well as use them to guide behavior [20]. According to the Emotional Intelligence Model [21], high levels of EI could make individuals immune from stress or less vulnerable to stress [22]. Mindfulness emphasizes awareness of one’s own experiences, feelings, and nonjudgmental attitudes [23]. The nonjudgmental component of mindfulness allows individuals to accurately perceive their own emotions, and the function of internal self-regulation of mindfulness can improve individuals’ ability to manage emotions. As a result, people who have a higher level of mindfulness may also have a higher level of emotional intelligence [24]. At the same time, people with higher levels of emotional intelligence tend to respond positively to stresses and challenges at work and in daily lives, which could reduce their feelings of stress and job burnout [25]. Because preschool teachers’ job characteristics cause them to experience more negative emotions than others, preschool teachers with low emotional competence are unable to perceive emotions in themselves and others, as well as self-regulate effectively, resulting in higher stress and poor interpersonal relationships, as well as higher job burnout [3]. Therefore, preschool teachers’ high level of mindfulness provides a foundation for their positive emotional competence to better cope with their professional pressure and job burnout. Some empirical studies have demonstrated that individuals’ mindfulness traits could indirectly affect their perceived stress and self-efficacy through EI [24,26,27]. A study of kindergarten teachers showed that mindfulness can negatively predict teachers’ psychological distress through emotional intelligence [28]. Similarly, studies on college students and adolescents suggested that mindfulness could affect their subjective well-being (e.g., negative emotions and life satisfaction) through EI [29,30]. A study on intensive care nurses also suggested that emotional intelligence mediated the relationship between mindfulness and occupational burnout [31]. Therefore, we hypothesize that EI would play a mediating role in the relations between preschool teachers’ mindfulness and job burnout.

### 1.3. Coping Style as a Mediator

According to Mindful Coping Model, when an individual’s assessment of the threat, harm, or loss of a particular event is beyond his/her ability, they will re-evaluate and attribute the event in order to adopt more appropriate coping strategies [32]. Mindfulness also helps individuals to be more aware of their experiences in the present moment, and when they can perceive their emotional feelings clearly, they tend to adopt appropriate ways to cope with external stresses and challenges in order to free themselves from negative emotions [33]. Coping is defined as a set of cognitive and behavioral strategies adopted to manage the internal and external demands in stressful situations [34]. Individuals may adopt a very different coping style when faced with the same type of pressure from work and life, which can be classified into positive coping style (e.g., seeking support, striving for change, problem-solving, etc.) and negative coping style (e.g., avoidance, venting, neglect, etc.) [35,36]. Empirical research on college students and mothers of special needs children had shown that individuals with a high level of mindfulness tend to use positive coping styles (e.g., proactive problem solving) when faced with stress, whereas those with a low level of mindfulness tend to use negative coping styles (e.g., avoidant approaches and escape from problems and stress) [33,37]. A study about paramedics indicated that mindfulness had a negative correlation to job burnout via positive and negative coping styles [14]. A study on healthcare professionals suggested that coping strategies mediated the link between mindfulness and emotional exhaustion [38]. So, a higher level of mindfulness in preschool teachers will help them adopt positive coping styles in the face of stress and challenges, experiencing less stress and negative emotions, and then experiencing a lower level of job burnout [39,40]. Accordingly, we hypothesized that the coping styles of preschool teachers would mediate the relationships between mindfulness and burnout.

### 1.4. Chain Mediator of Emotional Intelligence and Coping Style

In terms of the perspective of top-down regulation and control of individual behavior, mindfulness is closely related to the activation of specific brain regions in emotional processing, which provides the cognitive basis for individuals to achieve higher emotional intelligence [41]. Preschool teachers with higher EI tend to be able to adopt positive emotion regulation strategies and positive coping strategies to achieve a balanced state of mind and body when in the face of the pressure from work and life [42]. Some empirical studies have also shown that emotional intelligence was associated with coping styles. For example, studies on students and teachers revealed that emotional intelligence was positively related to problem-focused coping, and negatively related to negative emotional-focused coping style [43,44,45]. Different coping styles among preschool teachers will result in different emotional experiences and feelings in the face of pressure and challenges, which is one of the major factors contributing to job burnout [39]. As a result, we hypothesized that the relations between mindfulness and burnout would be serially mediated by emotional intelligence and coping style.

### 1.5. The Present Study

**Figure 1:**
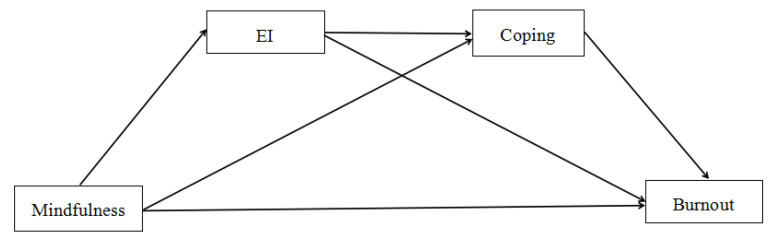
The proposed chain mediating model. EI = emotional intelligence.

According to the Mindful Emotion Regulation Model, Mindful Coping Model, and the existing literature research, the purpose of this study was to investigate the mediating roles of emotional intelligence and coping style in the association between mindfulness and preschool teachers’ job burnout in Chinese culture. We propose the following hypotheses based on existing research (see Figure 1). H1: Preschool teachers’ mindfulness is negatively related to job burnout. H2: Emotional intelligence would mediate the relationship between mindfulness and job burnout. H3a: Positive coping style would mediate the relationship between mindfulness and burnout. H3b: Negative coping style would mediate the relationship between mindfulness and burnout. H4a: The relationship between mindfulness and burnout would be serially mediated by emotional intelligence and positive coping style. H4b: The relationship between mindfulness and burnout would be serially mediated by emotional intelligence and negative coping style.

## 2. Materials and Methods

### 2.1. Participants

Participants were 394 kindergarten teachers (for children aged 3–6 years old), which consist 374 females and 20 males; 264 of them are from public kindergartens and 130 of them are from private kindergartens. These teachers also have a wide range of age (Range 21–58, Mean_age_ = 33.60 years, *SD* = 8.45 years), teaching experience (38.1% 5 years or less; 24.4% 6–10 years; 9.6% 11–15 years; 12.9% 16–20 years; 15% more than 21 years), and educational background (33.5% junior college; 59.4% bachelor degree; 7.1% master degree).

### 2.2. Measures

#### 2.2.1. Mindful Attention Awareness

Teachers’ mindfulness was measured using Mindful Attention Awareness Scale (MAAS) [46]. The MAAS consists of 15 items (e.g., I rush through activities without being really attentive to them) and participants rated each item on a six-point Likert scale (1 = almost always; 6 = almost never). Higher scores mean higher levels of teachers’ mindfulness. The Chinese version of MAAS used in this research had good psychometric properties [47]. Cronbach’s α coefficient of the scale in this study was 0.84.

#### 2.2.2. Emotional Intelligence

Teachers’ emotional intelligence was assessed using Emotional Intelligence Scale (EIS) [48]. The EIS consists of 19 items assessing teachers’ emotional intelligence on five sub-scales: perceiving emotion (4 items, e.g., By looking at people’s facial expressions, I recognize the emotions they are experiencing), use of emotion (3 items, e.g., When making decisions, I listen to my feelings to see if the decision feels right), understanding emotion (4 items, e.g., I have a rich vocabulary to describe my emotions), managing emotion (4 items, e.g., I have problems dealing with my feelings of anger), and social management (4 items, e.g., I know the strategies to make or improve other people’s moods). Participants rated each item on a five-point Likert scale (1 = Very inaccurate; 5 = Very accurate), with a high score meaning a high level of emotional intelligence. In this study, items were translated in Mandarin by one Chinese psychology professor and two doctoral students, and then independently back-translated to English to make sure there were no problems with the language. The Cronbach’s α coefficient of each subscale ranged from 0.69 to 0.80. There was good construct validity from the results of CFA as shown in χ^2^/df = 2.53, GFI = 0.92, IFI = 0.91, CFI = 0.91, RMSEA = 0.06.

#### 2.2.3. Teacher’s Coping Style

Teachers’ coping style was assessed using teacher self-rated Coping Style Scale (CSS) [49], which consists of 20 items and two sub-scales: positive coping style (12 items, e.g., change my mind and rediscover what’s important in your life) and negative coping style (8 items, e.g., time can change the situation, the only thing you can do is to wait). Participants completed each item on a four-point Likert scale (0 = never; 3 = often), with higher scores meaning a higher level of this coping style. This measure has been revised and demonstrated good psychometric properties in a Chinese context [36]. The Cronbach’s α coefficient of the two sub-scales was 0.81 and 0.71.

#### 2.2.4. Teacher’s Burnout

Teachers’ burnout was measured using the 3-factor Maslach Burnout Inventory Educator’s Survey (MBI-ES) [2]. The MBI-ES consists of 22 items assessing teachers’ burnout with three sub-scales: emotional exhaustion (9 items, e.g., I feel used up at the end of the workday), depersonalization (6 items, e.g., I feel I treat students as if they were impersonal objects), and personal accomplishment (7 items, e.g., I have accomplished many worthwhile things in this job). Participants rated each item on a seven-point Likert scale (1 = never; 7 = every day), with higher scores meaning a higher level of burnout during their work. This measure had been revised and demonstrated good psychometric properties in a Chinese context [50]. The Cronbach’s α coefficient of the three subscales in the study ranged from 0.80 to 0.90.

#### 2.2.5. Control Variables

Except for the main study variables, we also collected some demographic information (age and gender) of the teachers as control variables. Some studies also found that these demographic factors tend to determine the level of outcome variables [3,51]. So, in the following mediating effect analysis, we put teachers’ gender and age into control variables.

### 2.3. Procedure

Participants were recruited from the kindergartens in Shanghai and Anhui Province, China. We collected the data online. Specifically, we sent the link of the survey to the kindergarten director, who forwarded it to the teachers. The informed consent was on the first page of the online survey; if the teachers agreed to participate, they were directed to the survey; if they did not agree, they could click “Exit the Survey”. All participants signed an informed consent form, and all the teachers were informed of the purpose and details about this study before completing the four questionnaires, which included demographic information and scale items based on the filling instructions.

### 2.4. Analytic Strategy

We used SPSS22.0 (IBM, New York, NY, USA) to conduct the correlations, descriptive statistics, and the Cronbach’s α coefficient of each variables in this study. In addition, we also analyzed the relationships among the four variables via structural equation modeling (SEM) using AMOS 24.0 (IBM, New York, NY, USA). We used bootstrapping procedure 5000 resamples to assess the unconditional indirect effects, which are significant when 95% bias-corrected and accelerated confidence intervals (95% CI) do not contain zero. To evaluate the absolute model fit, we used the following fit indices: comparative fit index (CFI), incremental fit index (IFI), goodness of fit index (GFI), root mean square error of approximation (RMSEA), and χ^2^ test of significance. Close fit is indicated by CFI, IFI, and GLI > 0.90, and RMSEA < 0.08 [52].

## 3. Results

### 3.1. Preliminary Analyses

The means, SDS, and correlations of mindfulness, emotional intelligence, coping style, and burnout were displayed in Table 1. The results indicated that teachers’ background information (age and gender) was significantly correlated to the research variables. Among the research variables, mindfulness was significantly positively correlated to emotional intelligence (r = 0.46, *p* < 0.001) and positive coping style (r = 0.32, *p* < 0.001), and negatively correlated to negative coping style (r = −0.19, *p* < 0.01) and teachers’ burnout (r = −0.52, *p* < 0.001). Emotional intelligence was significantly positively correlated to positive coping style (r = 0.58, *p* < 0.001) and negatively correlated to teachers’ burnout (r = −0.52, *p* < 0.001). Positive coping style was negatively correlated to teachers’ burnout (r = −0.46, *p* < 0.001). Negative coping style was significantly positively correlated to teachers’ burnout (r = −0.24, *p* < 0.01).

### 3.2. Measurement Model

Regarding measurement models [53], the full measurement model was initially tested, this model included all the variables, and all the items loaded on their theoretical constructs, and generated a good fit (χ^2^/df = 2.97, CFI = 0.95, IFI = 0.95, GFI = 0.95, RMSEA = 0.07). We then performed a Harman’s single-factor test; this test involves a CFA in which all variables are allowed to load onto one general factor, and the one-factor model showed a poor fit to the data (χ^2^/df = 7.86, CFI = 0.52, IFI = 0.52, GFI = 0.65, RMSEA = 0.15). Furthermore, the full measurement model fitted the data significantly better than the single-factor model (Δχ^2^ = 640.55, Δdf = 7, *p* < 0.001).

### 3.3. Direct Effects among Variables

We conducted a series of direct effect analyses among study variables prior to the mediation analysis. The findings revealed that, while mindfulness in preschool teachers had a significantly negative effect on burnout (β = −0.64, *p* < 0.001) and negative coping (β = −0.19, *p* < 0.001), mindfulness had a significantly positive effect on EI (β = 0.52, *p* < 0.001) and positive coping (β = 0.32, *p* < 0.001). EI had a significantly negative effect on burnout (β = −0.53, *p* < 0.001) and a significantly positive effect on positive coping (β = 0.65, *p* < 0.001), but no significant effect on negative coping (β = −0.05, *p* > 0.05). Positive coping had a significantly negative effect on burnout (β = −0.47, *p* < 0.001), while negative coping had a significantly positive effect on burnout (β = 0.27, *p* < 0.001).

### 3.4. Mediation Analyses

The mediating effect of emotional intelligence and coping style between teachers’ mindfulness and burnout was conducted. Teachers’ gender and age were put into control variables. According to the results of correlations in Table 1, emotional intelligence was not significantly related to negative coping style. So, in the following mediation analysis, we did not include the path from emotional intelligence to negative coping style, and then the Hypothesis 4b was not supported; the statistics of Hypothesis 4b was not included in Table 2. The fitting index of this mediating model was good (χ^2^/df = 2.97, GFI = 0.95, IFI = 0.95, CFI = 0.95, RMSEA = 0.07).

**Figure 2:**
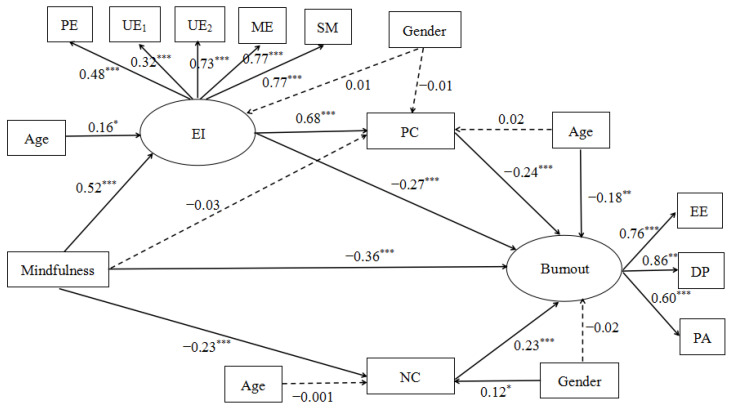
The chain mediation model. Note: * *p* < 0.05, ** *p* < 0.01, *** *p* < 0.001. EI = emotional intelligence, PC = positive coping style, NC = negative coping style, PE = perceiving emotion, UE_1_ = use of emotion, UE_2_ = understanding emotion, ME = managing emotion, SM = social management, EE = emotional exhaustion, DP = depersonalization, PA = personal accomplishment.

The results (see Figure 2 and Table 2) showed that, in Hypothesis 1, mindfulness had a significant negative effect on burnout (β = −0.36, *p* < 0.001). In Hypothesis 2, there was a significant mediation effect of mindfulness on burnout through emotional intelligence. The indirect effect was −0.14, and the 95% confidence interval (CI) = [−0.25, −0.07] does not contain zero. In Hypothesis 3a, the indirect effect of positive coping style between mindfulness and burnout was not significant, and the 95% confidence interval (CI) = [−0.02, 0.03]. In Hypothesis 3b, there was a significant mediation effect of mindfulness on burnout through negative coping style, the indirect effect was −0.06, and the 95% confidence interval (CI) = [−0.11, −0.03] does not contain zero. In Hypothesis 4a, emotional intelligence and positive coping style have a chain mediating effect between mindfulness and job burnout, the indirect effect was −0.09, and the 95% confidence interval (CI) = [−0.14, −0.05] does not contain zero.

## 4. Discussion

This study investigated the relationship between Chinese preschool teachers’ mindfulness and burnout, and the mediating roles of emotional intelligence and coping style. The results revealed that preschool teachers’ mindfulness was negatively related to their burnout; emotional intelligence and negative coping style mediated the relationship between mindfulness and burnout independently; and emotional intelligence and positive coping style played a chain mediating role between mindfulness and burnout.

### 4.1. Relations between Mindfulness and Burnout

The current study found that preschool teachers’ mindfulness was negatively associated with their job burnout, which was consistent with the previous research [11]. If preschool teachers can often pay attention to their own current emotion and physical experience during their daily life and work, this can help them better to perceive the emotional feelings, cope with stress, deal with interpersonal relationships, improve their professional well-being and life satisfaction, and then alleviate job burnout [14]. Many schools and educational institutions also implement mindfulness-based intervention to reduce their employee’s psychological pressure and alleviate their feelings of job burnout [17,54]. Furthermore, mindfulness can also be integrated into preschool teachers’ daily life and work, maintaining the state of mindfulness and paying attention to the present moment can improve the quality of life and work efficiency, and, consequently, alleviating their job burnout [55].

### 4.2. The Independent Mediating Role of Emotional Intelligence and Coping Style

The present study found that emotional intelligence independently mediated the relationship between teachers’ mindfulness and job burnout. Specifically, a higher level of mindfulness was associated with a higher level of emotional intelligence, then serving to mitigate preschool teachers’ feeling of job burnout. Charoensukmongkol (2014) also found that mindfulness can affect perceived stress through emotional intelligence [24]. According to the basic biological research, mindfulness can activate the prefrontal cortex and left amygdala of the brain during emotional processing [41], and these brain regions have the functions of recognizing and regulating emotions as well as emotional memory, which can help individuals to better perceive and regulate emotions and reduce psychological stress [56]. Moreover, the Emotion Regulation Model of mindfulness also holds that, when we accept the problems and challenges during daily life and work without negative evaluations, it can often improve emotional adaptability and reduce the level of burnout [19]. Findings from the current study also revealed that preschool teachers’ negative coping style mediated the relations between mindfulness and job burnout, which was similar to the findings of previous studies [14]. Moreover, these findings were consistent with Mindful Coping Model, which suggests that, when faced with a stressful and challenging situation, individuals will reevaluate and attribute the event in order to adopt more appropriate strategies, and then reduce the harm to the individual caused by the stress [32].

In addition, the results of this study did not find the mediating effect of positive coping style between mindfulness and burnout, which was inconsistent with research Hypothesis 3a. It is now well established from a study on college students that mindfulness was significantly associated with negative coping style (avoidant coping) rather than positive coping style [57]. Possible reasons are that mindfulness has more emphasis on individuals’ awareness of their experiences and being nonjudgmental, while negative coping style means ignoring and avoiding problems instead of taking effective approaches to solving them. In this regard, preschool teachers with a low level of mindfulness are more likely to adopt negative coping style when faced with stressful situations for avoiding these problems provisionally. Thus, this study found that mindfulness is not a significant predictor of positive coping style.

### 4.3. The Chain Mediating Effect of Emotional Intelligence and Coping Style

Consistent with Hypothesis 4a, the result of this study shows that there was a chain mediating role of emotional intelligence and positive coping style between mindfulness and burnout. Research related to mindfulness and emotions has shown that mindfulness allows individuals to detect the cues related to negative emotions (e.g., anger) in time, and then respond more appropriately and avoid undesirable consequences of automated reactions [58]. Thus, mindfulness of preschool teachers not only provides the basis and possibility to better recognize and perceive the emotions of themselves and others, effectively managing emotions, but also enhances their emotional intelligence, helping them to adopt effective ways to cope with challenges and stress. Preschool teachers with a higher level of emotional intelligence are more likely to adopt positive coping styles in the face of stress; instead of relocating their negative emotions, they focus on the problem and try to find ways to solve it or look for social support [43]. Such a positive coping style allows them to maintain a positive emotional state from time to time and to find strategies to solve problems quickly and appropriately, all of which can reduce their feelings of stress and avoid job burnout.

Nevertheless, this research did not find a chain mediating role of emotional intelligence and negative coping style between mindfulness and burnout, which is inconsistent with Hypothesis 4b. Some existing studies about teachers have also proposed that emotional intelligence can positively predict positive coping style rather than negative coping style [42,43]. Emotional intelligence emphasizes individuals’ ability to perceive, manage, regulate, and express emotions; therefore, preschool teachers with a high level of emotional intelligence are more likely to adopt positive coping style to solve problems when faced with difficult situations rather than negative coping style, such as avoidance, neglect, or doing nothing. Therefore, the present research found that emotional intelligence was not a significant predictor of negative coping style, but could significantly predict positive coping style. This finding further clarified the Mindful Emotion Regulation Model; mindfulness can improve one’s EI, and then make them adopt more positive coping strategies, but a low level of mindfulness can directly lead individuals to take more negative coping styles.

### 4.4. Implications and Limitations

This study explored the relationship between preschool teachers’ mindfulness and job burnout from the perspective of individual factors, and found the serial mediating effects of emotional intelligence and positive coping styles between mindfulness and job burnout.

The study’s findings made significant theoretical contributions. First of all, the evidence revealed the mechanism of mindfulness on job burnout among preschool teachers, as well as the chain mediation effects among the four variables. However, few previous studies have investigated the combined effect of individual characteristics on job burnout. As a result, this study filled a gap in previous research on the factors that influence teacher burnout. Secondly, according to the findings of this study, which integrated two theoretical models: “Mindful Emotion Regulation Model” and “Mindful Coping Model”, preschool teachers’ high level of mindfulness can improve individuals’ emotional intelligence, which can then contribute to teachers’ positive coping and reduce burnout. As a result, the findings broadened the scope of theoretical models related to mindfulness. Furthermore, the study’s findings had significant practical implications. To begin with, the findings reminded us that individual characteristics of preschool teachers (such as mindfulness, emotional intelligence, and coping style) are important influencing factors of burnout, and we should pay more attention to these factors at work and in daily life. Secondly, this study pointed the way forward for future psychological interventions for preschool teachers in China. Mindfulness-based intervention programs can effectively reduce teacher burnout. Mindfulness practices can also improve teachers’ emotional competence and encourage them to use more positive coping strategies when faced with challenges, ultimately improving their happiness at work and in life.

The current study also had several limitations. Firstly, our study was a correlational design, which limits our ability to explore causal links and the direction of effects between the constructs. In the future, a longitudinal research design could be used to explore the mediation effects. Secondly, the factors influencing burnout are multiple, and the current study mainly focused on individual factors. So, the environmental factors can be included in the model in future studies to examine the effects of individual and environmental factors on burnout. Finally, due to the large gender differences in the sample, the gender differences among variables could not be examined in this study, and the relative balance of sample sizes of different gender could be considered in future studies.

## 5. Conclusions

Overall, this study found a link between preschool teachers’ mindfulness and job burnout. Preschool teachers with a high level of mindfulness could perceive, understand, use, and manage their emotions better, and tended to use positive coping strategies when faced with challenges, resulting in less burnout. A lack of mindfulness, on the other hand, would exacerbate preschool teachers’ job burnout through emotional intelligence and coping style.

## Figures and Tables

**Table 1:** Correlations and descriptive statistics of measured variables.

	*M*	*SD*	1	2	3	4	5	6	7
1. Gender	−	−	1						
2. Age	33.60	8.45	0.10	1					
3. Mindfulness	4.53	0.74	−0.04	−0.11 *	1				
4. Emotional intelligence	3.51	0.47	0.02	0.12 *	0.46 ***	1			
5. Positive coping style	2.18	0.45	−0.01	0.12 *	0.32 ***	0.58 ***	1		
6. Negative coping style	1.36	0.52	−0.12 *	0.01	−0.19 **	−0.02	0.15 **	1	
7. Burnout	2.66	0.84	0.03	−0.16 **	−0.52 ***	−0.52 ***	−0.46 ***	0.24 ***	1

Note: 0 = male, 1 = female; * *p* < 0.05, ** *p* < 0.01, *** *p* < 0.001.

**Table 2:** Bootstrapping indirect effects and 95% confidence interval for the mediation model.

Hypothesis Model	β	SE	95% CI	
			LL	UL
H1: Mindfulness—Burnout	−0.36 ***	0.05	−0.58	−0.23
H2: Mindfulness—EI—Burnout	−0.14 **	0.04	−0.25	−0.07
H3a: Mindfulness—Positive Coping—Burnout	0.01	0.01	−0.02	0.03
H3b: Mindfulness—Negative Coping—Burnout	−0.06 *	0.02	−0.11	−0.03
H4a: Mindfulness—EI—Positive Coping—Burnout	−0.09 *	0.03	−0.14	−0.05

Note: * *p* < 0.05, ** *p* < 0.01, *** *p* < 0.001.

## Data Availability

The data presented in this study are available on request from the corresponding author. The data are not publicly available due to privacy.
